# Structural insights into antibody-based immunotherapy for hepatocellular carcinoma

**DOI:** 10.1186/s44342-024-00033-0

**Published:** 2025-01-20

**Authors:** Masaud Shah, Muhammad Hussain, Hyun Goo Woo

**Affiliations:** 1 Department of Physiology, Ajou University School of Medicine, Suwon, 16499 Republic of Korea; 2 Department of Biomedical Science, Graduate School, Ajou University, Suwon, 16499 Republic of Korea; 3 Ajou Translational Omics Center (ATOC), Research Institute for Innovative Medicine, Ajou University Medical Center, Suwon, Republic of Korea

**Keywords:** Antibodies, Bispecific antibodies, Immunotherapy, Hepatocellular carcinoma, HCC

## Abstract

Hepatocellular carcinoma (HCC) is one of the most common types of primary liver cancer and remains a leading cause of cancer-related deaths worldwide. While traditional approaches like surgical resection and tyrosine kinase inhibitors struggle against the tumor’s immune evasion, monoclonal antibody (mAb)-based immunotherapies have emerged as promising alternatives. Several therapeutic antibodies that counter the immunosuppressive tumor microenvironment have demonstrated efficacy in clinical trials, leading to FDA approvals for advanced HCC treatment. A crucial aspect of advancing these therapies lies in understanding the structural interactions between antibodies and their targets. Recent findings indicate that mAbs and bispecific antibodies (bsAbs) can target different, non-overlapping epitopes on immune checkpoints such as PD-1 and CTLA-4. This review delves into the epitope-paratope interactions of structurally unresolved mAbs and bsAbs, and discusses the potential for combination therapies based on their non-overlapping epitopes. By leveraging this unique feature, combination therapies could enhance immune activation, reduce resistance, and improve overall efficacy, marking a new direction for antibody-based immunotherapy in HCC.

## Introduction

Hepatocellular carcinoma (HCC) is the most common type of primary liver cancer and a serious public health concern [1]. According to 2022 global cancer statistics, HCC is the sixth most abundant cancer in 185 countries and fourth leading cause of cancer-related deaths worldwide [2, 3]. The risk of HCC is strongly linked to chronic liver diseases, with cirrhosis, non-alcoholic fatty liver disease, and chronic hepatitis B and C (HBV and HCV) infections being the primary contributing factors [4, 5]. HCC is particularly high in East Asia and sub-Saharan Africa, where HBV and HCV infections are common [5, 6].

The standard treatment modalities for HCC are multifaceted but face limitations. Surgical resection and liver transplantation are potential treatments for early-stage HCC, but only a small number of patients are eligible due to advanced disease or underlying hepatic dysfunction [7]. For intermediate-stage disease, locoregional therapy such as trans-arterial chemoembolization and radiofrequency ablation is frequently utilized [8]. For advanced HCC, systemic treatments, tyrosine kinase inhibitors (TKIs) like sorafenib and lenvatinib, are used [9, 10]. Nonetheless, resistance to therapy remains a serious challenge, and the overall survival benefits of these drugs are modest [11–13].

**Fig. 1:**
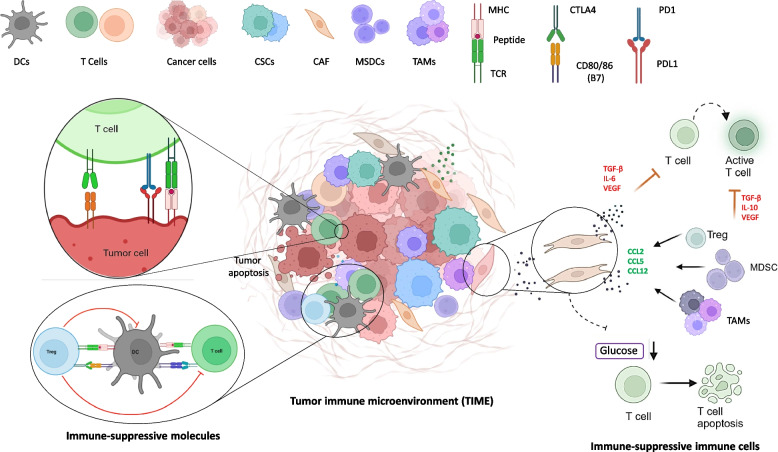
Brief overview of the immune evasion mechanisms in hepatocellular carcinoma (HCC). Various ways through which HCC manipulates the tumor immune microenvironment (TIME) and eludes immune monitoring are demonstrated. Important immune-suppressive molecules that lead to the suppression of T cell activation and induction of T cell exhaustion are increased on tumor cells and infiltrating immune cells. These molecules include PD-L1/PD-1, CTLA-4/B7, and TIM-3/Galectin-9. TIME is additionally distinguished by a significant influx of immune-suppressive cells, including as myeloid-derived suppressor cells (MDSCs), regulatory T cells (Tregs), and tumor-associated macrophages (TAMs), which secrete cytokines such as VEGF, IL-10, and TGF-β. These cytokines promote angiogenesis, tumor development, and immune suppression. Furthermore, extracellular matrix elements and angiogenic factors like VEGF are secreted by cancer-associated fibroblasts (CAFs) and TAMs in the TIME, which helps to physically exclude immune cells from the tumor core and strengthens the immunosuppressive environment. CAFs also release additional immunosuppressive cytokines and chemokines, such as IL-6 and CXCL12, which further suppress T cell infiltration and enhance the recruitment of other immunosuppressive cells like Tregs and TAMs. By reducing glucose, HCC cells also provide an environment that is metabolically unfavorable, which inhibits effector T cell function and fosters an immunosuppressive phenotype

The immune system plays a pivotal role in the development and progression of HCC [14]. Chronic liver inflammation, driven by viral infections or metabolic stress, creates an environment favorable for immune evasion, tumor growth, and metastasis [15–17]. HCC tumors can evade immune monitoring through a variety of strategies (Fig. 1). One of the primary strategies is the upregulation of immune checkpoint molecules, such as programmed cell death ligand-1 (PD-L1), on tumor and stromal cells [18, 19]. PD-L1 binds to programmed cell death proteins-1 (PD-1) receptors on active T cells, leading to T cell exhaustion, decreased cytokine production, and impaired cytotoxic activity [18]. This interaction not only prevents T cell-mediated destruction of tumor cells, but also contributes to an overall decrease in anti-tumor immunity, allowing the tumor to proliferate and metastasize unchecked [17]. Furthermore, HCC generates a profoundly immunosuppressive TME, marked by a high infiltration of regulatory T cells (Tregs), myeloid-derived suppressor cells (MDSCs), and tumor-associated macrophages (TAMs) [20, 21]. These cells release a spectrum of immunosuppressive cytokines, including transforming growth factor-beta (TGF-β) and interleukin-10 (IL-10), which further diminish the activity of effector T cells and promote tumor tolerance (Fig. 1) [22]. Moreover, cancer-associated fibroblasts (CAFs) and TAMs release extracellular matrix components and angiogenic factors such as vascular endothelial growth factor (VEGF), which promote tumor vascularization and provide a physical barrier that hinders immune cell infiltration into the tumor core [23, 24]. These complex interactions between tumor cells and the immune microenvironment make HCC challenging to treat with conventional therapies alone.

Given these complex immune evasion mechanisms, immunotherapy has emerged as a promising strategy for the treatment of HCC (Table 1). Antibody-based therapies target immune checkpoints and aim to strengthen the body’s natural immune response against cancer cells. Here, we provide a brief overview of advancements in antibody-based immunotherapies for HCC, with a particular focus on structural insights into the interactions between therapeutic antibodies and their targets. Understanding these molecular interactions is essential for comprehending the mechanisms that drive the efficacy and therapeutic potential of these treatments.

**Table 1:** Antibodies against immune suppressive checkpoint molecules

Antibody	Target	PDB ID	Indications	NCT
Pembrolizumab	PD-1	5JXE	Melanoma (approved), HCC (approved)	NCT02702414 (HCC) NCT02576574
Nivolumab	PD-1	5WT9	Melanoma (approved), HCC (approved ipilimumab + nivolumab)	NCT01658878 (HCC) NCT01721772
Dostarlimab	PD-1	7WSL	Endometrial cancer (approved), HCC (Trials)	NCT02215282, NCT03680508 (HCC)
Cemiplimab	PD-1	7WVM	ACSSC (approved), HCC (trials)	NCT02760498, NCT03916627 (HCC)
Camrelizumab	PD-1	7CU5	HCC (trials)	NCT03764293 (HCC)
Sintilimab	PD-1	PLAbDAb	HCC (trials)	ChiCTR2000037655 (HCC) NCT03794440 (HCC)
Retifanlimab	PD-1	PLAbDAb	Solid tumors, HCC (none)	NCT03679767
Spartalizumab	PD-1	PLAbDAb	HCC (trials)	NCT02795429 (HCC) NCT02325739 (HCC)
Atezolizumab	PD-L1	5X8L	Bladder cancer (approved), HCC (approved with bevacizumab as combo)	NCT02302807, NCT03434379 (HCC)
Durvalumab	PD-L1	5X8M	Bladder cancer (approved), HCC (approved with tremelimumab as combo)	NCT01693562 NCT03298451 (HCC)
Avelumab	PD-L1	5GRJ	Merkel cell carcinoma (approved), Adv HCC (trials)	NCT02155647, NCT03389126 (HCC)
Adebrelimab	PDL-1	PLAbDAb	SCLC, HCC (trials)	NCT03711305 NCT06405061 (HCC)
Cosibelimab	PDL-1	PLAbDAb	SCC (trials), HCC (none)	NCT03212404
Sugemalimab	PD-L1	PLAbDAb	Multiple (including HCC)	NCT03312842 (HCC)
Ipilimumab	CTLA-4	5TRU	HCC (approved ipilimumab + nivolumab), Metastatic melanoma (approved)	NCT01658878 (HCC) NCT00094653
Tremelimumab	CTLA-4	5GGV	HCC (approved with durvalumab as combo)	NCT02519348 (HCC) NCT03298451 (HCC)
Hl32	CTLA-4	6XY2	Cancers (pre-clinical)	PMID: 31,267,017
Relatlimab	LAG-3	–	Melanoma (approved), HCC (trials)	NCT03470922 NCT05337137 (HCC)
Cobolimab	TIM-3	8GSI	NSCLC (trials), HCC (trials)	NCT03708328 NCT03680508 (HCC)
Onvatilimab	VISTA	PLAbDAb	Solid tumors (trials), HCC (none)	NCT02671955 NCT04475523
Enoblituzumab	B7-H3/B7-H4	PLAbDAb	Prostate cancer (trials), HCC (none)	NCT02923180

## Antibodies against immune suppressive checkpoints molecules

Immune checkpoint proteins are key modulators that tumors exploit to suppress immune responses and evade destruction [25]. The overexpression of these molecules within the TME dampens T cell function and enables cancer cells to thrive [26]. To combat this, antibody-based immunotherapies have lately emerged as a radical approach to revive the effective tumor immune microenvironment (TIME) [27]. In addition to PD-1/PD-L1 expression in TIME, CTLA-4, expressed by Tregs, binds to B7-1 (CD80) and B7-2 (CD86) on antigen-presenting cells (APCs), sending inhibitory signals to suppress T cell activity (Fig. 1) [28]. In the absence of CTLA-4, CD28, which also binds to B7-1 and B7-2, delivers activating signals to promote T cell responses [28]. This competition between CTLA-4 and CD28 for B7 molecules reduces T cell responses, as CTLA-4 is highly expressed within the HCC TIME [29]. Due to the capability of cancers to exploit the suppression of T cell activation, CTLA-4 has remained a prime candidate for antibody treatments (Table 1). Antibodies like ipilimumab and tremelimumab—with the latter being FDA-approved specifically for HCC [30]—target CTLA-4 to enhance T cell responses [31, 32].

Lymphocyte activation gene 3 (LAG-3) binds to MHC II molecules on APCs to limit T cell activation and proliferation [33]. Its expression is linked to T cell fatigue in the TME, and T cell responses against malignancies are strengthened when LAG-3 is inhibited [34]. In addition, T-cell exhaustion is largely promoted by T-cell immunoglobulin and mucin domain 3 (TIM-3), which is expressed on T cells and APCs. It binds to a variety of ligands, such as galectin-9, phosphatidylserine, and CEACAM1, causing inhibitory signals that reduce T cell activation [35]. Antibodies like cobolimab block TIM-3 and recover T cells, allowing them to mount a more effective response against the tumor [36, 37]. V-domain Ig suppressor of T cell activation (VISTA) prevents T cell activation and promotes immunological tolerance inside the TME [38]. VISTA is currently being evaluated in numerous clinical trials targeting different cancers [39]; however, its role remains underexplored in HCC, making it a compelling target for future research and potential therapeutic development. To disrupt these processes and reestablish potent anti-tumor immunity, antibody-based immunotherapies—in particular, ICIs—have been created (Table 1). A comprehensive knowledge of the specific mechanisms by which these antibodies provide their therapeutic advantages is necessary to optimize their clinical utilization [40]. The following section explores the mechanisms of antibody-receptor interactions for immune checkpoint targeting antibodies, focusing on those that are FDA-approved or under clinical investigation for HCC. It emphasizes how understanding these interactions can enhance treatment efficacy, especially in the context of combination therapies.

## Structural insights into the immunotherapeutic antibodies

### PD-1 targeting antibodies in HCC

Till now, two PD-1-targeting antibodies, pembrolizumab [41] and nivolumab [42], have received FDA approval for HCC under specific conditions, marking significant progress in PD-1-targeted therapy for liver cancer (Table 1). Pembrolizumab was initially granted accelerated approval in 2018 for patients with HCC who had been previously treated with sorafenib, though this indication was subsequently revised in 2021 [43]. Meanwhile, nivolumab, in combination with ipilimumab, has been approved for HCC patients previously treated with sorafenib, based on the CheckMate-040 study [31]. For some antibodies, detailed crystal structures are available, providing a foundational understanding of their PD-1 binding mechanisms. However, for antibodies like spartalizumab [44], retifanlimab [45], sugemalimab [46], and sintilimab [47], where no crystal structures are resolved, we used AlphaFold 3 [48] to predict their binding conformations with PD-1. Structural data were further annotated using IGMT annotations of the CDR loops, offering precise interaction profiles for these antibodies [49].

**Fig. 2:**
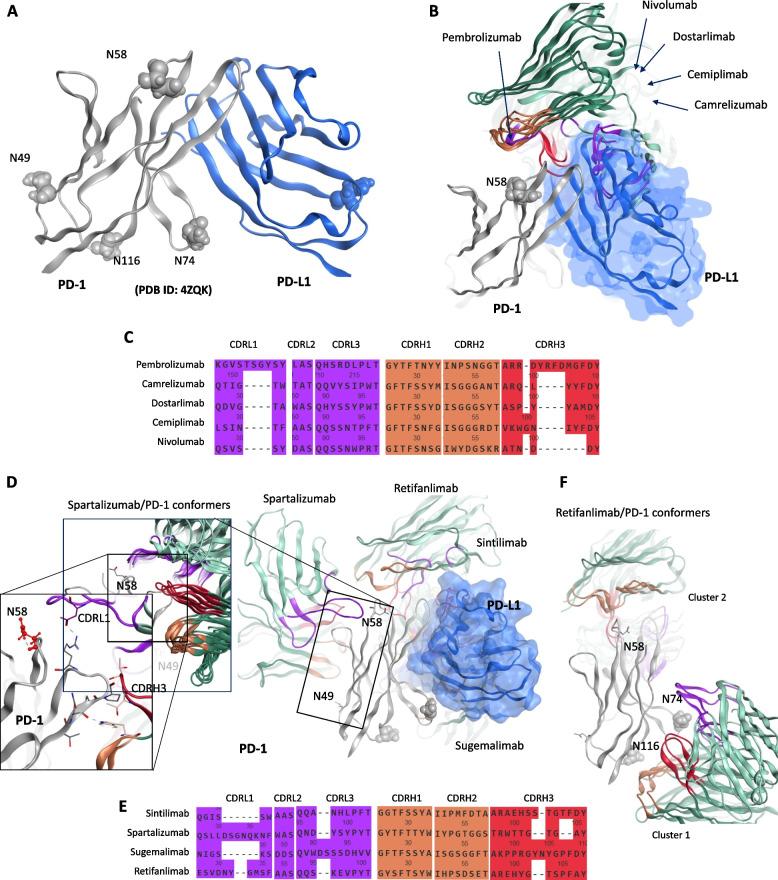
PD-1/PD-L1 interface and binding mechanism of PD-1 inhibiting monoclonal antibodies (mAbs). **A** Illustration of the PD-1/PD-L1 interaction interface. **B** Superimposition of PD-1 binding mAbs on a single PD-1 molecule, with corresponding PDB IDs listed in Table 1. **C** Alignment of the complementarity-determining regions (CDRs) from the mAbs presented in panel **B**. **D** Superimposition of PD-1 binding mAbs on a single PD-1 molecule, constructed using AlphaFold 3. **E** Alignment of the CDRs from the mAbs depicted in panel **D**. **F** Identification of two non-overlapping epitopes for retifanlimab, one of which overlaps with nivolumab (*panel ****B***), along with new glycosylation sites at N74 and N116

The interaction between PD-1 and PD-L1 involves a specific interface on their IgV domains, similar to antigen-receptor interactions in immune cells [50]. PD-1 and PD-L1 complex forms an Fv-like complex similar to antibodies and T cell receptors (Fig. 2A). Key residues on PD-1/PD-L1 interface are listed in Table 2. In addition, PD-1 has N-linked glycosylation sites at residues N49, N58, N74, and N116, while PD-L1 has one at residue N35. These glycosylations do not interfere with the binding interface between PD-1 and PD-L1 (Fig. 2A); however, some studies suggest that glycosylation of PD-1, particularly at N58, enhances its interaction with camrelizumab [51]. Almost all PD-1-targeting antibodies inhibit the PD-L1 binding site on PD-1, though they are not specifically engineered to differentiate between glycosylated and non-glycosylated forms of PD-1. To investigate their inhibitory mechanisms, we superimposed all structurally resolved PD-1/mAb complexes onto the PD-1/PD-L1 interaction site (Fig. 2B). We observed that, except for pembrolizumab, the variable light (VL) and variable heavy (VH) chains of the four monoclonal antibodies overlapped significantly with each other, with the VL chains effectively blocking the PD-L1 binding site on PD-1. In the case of pembrolizumab, the VH chain, rather than the VL, competes directly with PD-L1 at the binding interface (Fig. 2B). Moreover, the CDRs of all these antibodies were located far from the N58 glycosylation site on PD-1. This indicates that glycosylation at N58 may have an allosteric effect on the efficacy of camrelizumab, as their key binding regions do not interact with this site. Despite the lack of design specificity for glycosylated PD-1, the antibodies appear to remain highly effective in their role as PD-L1 inhibitors. We also observed that the CDRL1 and CDRH3 of pembrolizumab are lengthier than that of other candidates; however, their other CDRs are equal in length but variable to certain degree (Fig. 2C).

**Table 2:** Interface of PD-1/PD-L1 (PDB ID: 4ZQK). This table corresponds Fig. 2A, showing detailed interface of the PD1/PDL1 complex

Bond	PD-1	PD-L1	Energy	Dist
IH	Glu136	Arg113	− 32.1	2.96
IH	Asp77	Lys124	− 21.96	2.7
H	Lys78	Phe19	− 11.1	2.81
H	Lys78	Ala121	− 7.8	2.89
H	Gln75	Arg125	− 7.5	2.89
IH	Glu84	Ala18	− 5.21	3.18
H	Gln75	Asp26	− 3.8	2.79
H	Asn66	Ala121	− 2.9	2.88
H	Glu84	Phe19	− 2.7	3.12
H	Ala132	Gln66	− 1.5	2.84
H	Lys131	Gln66	− 0.7	3.29

Unit for energy is kcal/mol; *I* ionic bonds; *H* hydrogen bonds

For the other four mAbs selected in this study, we used AlphaFold 3 to understand their binding mechanism onto PD-1. Sintilimab and sugemalimab clustered around the PD-L1 binding interface of PD-1, indicating that they likely compete with PD-L1 for binding (Fig. 2D). In contrast, spartalizumab and retifanlimab adopted distinct binding conformations. Remarkably, all five conformers of spartalizumab consistently clustered at a position on PD-1 harboring the N58 and N49 glycosylation sites, opposite to the PD-L1 binding site (Fig. 2B). A closer inspection revealed that these glycosylation sites remained distant from the antibody’s CDRs, particularly CDRH3. Although the AlphaFold 3 model was generated with high confidence, this raise concerns regarding spartalizumab’s ability to inhibit PD-L1, as it does not appear to compete directly at the PD-1/PD-L1 interaction interface. One hypothesis could involve allosteric conformational changes induced by spartalizumab binding, potentially altering the PD-1 structure and indirectly affecting PD-L1 binding. In addition, we found that CDRL1 of the spartalizumab, which establish contact with PD-1, is unusually longer than on that of other mAbs (Fig. 2E). However, this will require rigorous experimental validation to confirm the proposed hypothesis. Retifanlimab exhibited two binding positions on PD-1, with one overlapping with nivolumab’s binding site and another near the N74 and N116 glycosylation sites (Fig. 2F). Given that these glycosylation sites could potentially interfere with retifanlimab’s binding, and considering that this antibody has been shown to block PD-L1 binding effectively [45], we propose that the first conformation (overlapping with nivolumab) is more likely to be its functional state. Overall, both AlphaFold-modeled structures and available X-ray models suggest that most PD-1-binding antibodies overlap at the PD-L1 binding site on PD-1. However, spartalizumab appears to target a unique site, offering a potential rationale for combining spartalizumab with other PD-1-targeting antibodies to achieve synergistic effects. These insights could pave the way for advanced treatment strategies in HCC by enhancing anti-tumor efficacy through combination therapy.

### PD-L1 targeting antibodies in HCC

**Fig. 3:**
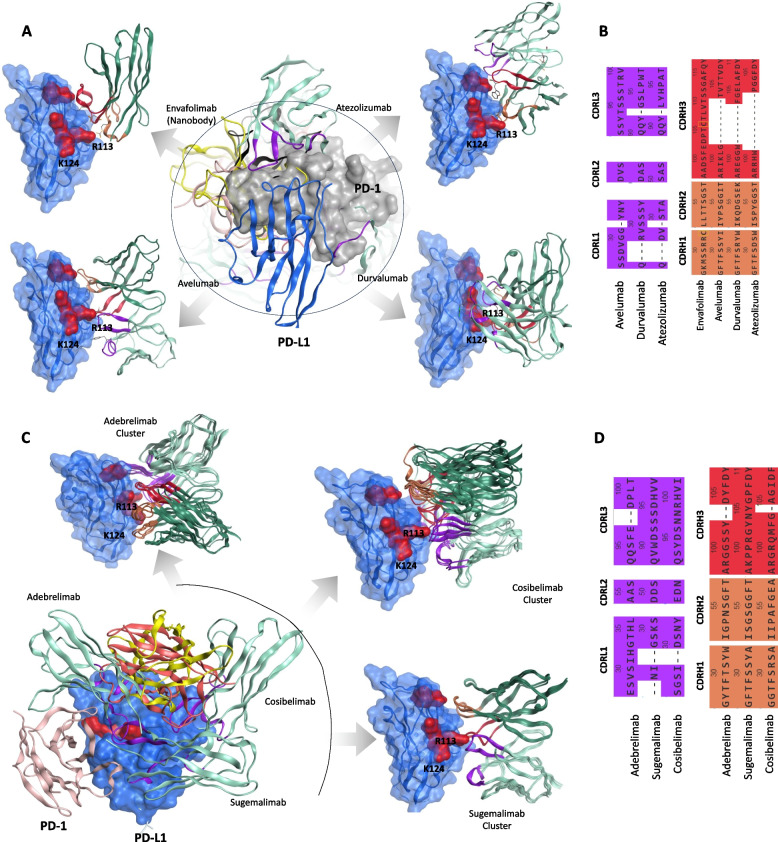
Binding mechanism of PD-L1 inhibiting monoclonal antibodies (mAbs). **A** PD-L1 binding mAbs are superimposed on a single PD-1 molecule, with corresponding PDB IDs listed in Table 1. PD-1 binding basic residues R113 and K124 are shown in as red surfaces. **B** Alignment of the complementarity-determining regions (CDRs) from the mAbs presented in panel **A**. **C** Superimposition of PD-L1 binding mAbs, constructed using AlphaFold 3. **D** Alignment of the CDRs from the mAbs depicted in panel **C**

PD-L1 inhibiting mAb, atezolizumab, in combination with the anti-VEGF antibody bevacizumab, has received FDA approval for unresectable HCC based on the IMbrave150 trial [52]; however, no PD-L1-targeting antibody has been approved as standalone treatment for HCC (Table 1). This combination therapy has demonstrated significant improvements in overall survival and progression-free survival compared to sorafenib, making it the preferred option for many patients [53]. The interface of the PD-1/PD-L1 complex reveals that basic residues R113 and K124 of PD-L1 form strong electrostatic interactions with the acidic residues E136 and D77 on PD-1, respectively (Table 2). Since these residues are essential for stabilizing the PD-1/PD-L1 interaction [54], antibodies targeting PD-L1 need to block this interface to disrupt the interaction. To investigate this, we superimposed the reported PD-L1/mAb structures and evaluated their inhibitory mechanisms (Fig. 3A). The VH domain of the envafolimab nanobody and three other mAbs fully occupy the R113 and K124 residues of PD-L1, preventing PD-1 from accessing these residues (Fig. 3A, *middle panel*). CDR annotation of these mAbs indicate that CDRH3 of the envafolimab nanobody is unusually longer than the CDRH3 of the rest of mAbs (Fig. 3B).

In addition, we modeled the structures of adebrelimab, cosibelimab, and sugemalimab and investigated their binding to PD-L1 using AlphaFold 3, as their structure has not been resolved experimentally. These models showed that the binding conformers of all three antibodies were highly specific, with an overlapping epitope on PD-L1 that includes the crucial R113 and K124 residues (Fig. 3C). CDR alignment of these mAbs suggest substantial differences (Fig. 3D). Unlike spartalizumab, which binds PD-1 at a distinct site compared to other PD-1 antibodies, all seven PD-L1 antibodies investigated here bound to an overlapping epitope on PD-L1, particularly at R113 and K124 residues. These findings suggest that the targeting of R113 and K124 is a common strategy among PD-L1 inhibitors. This insight could guide the development of future therapies for HCC.

### CTLA-4 targeting antibodies in HCC

**Fig. 4:**
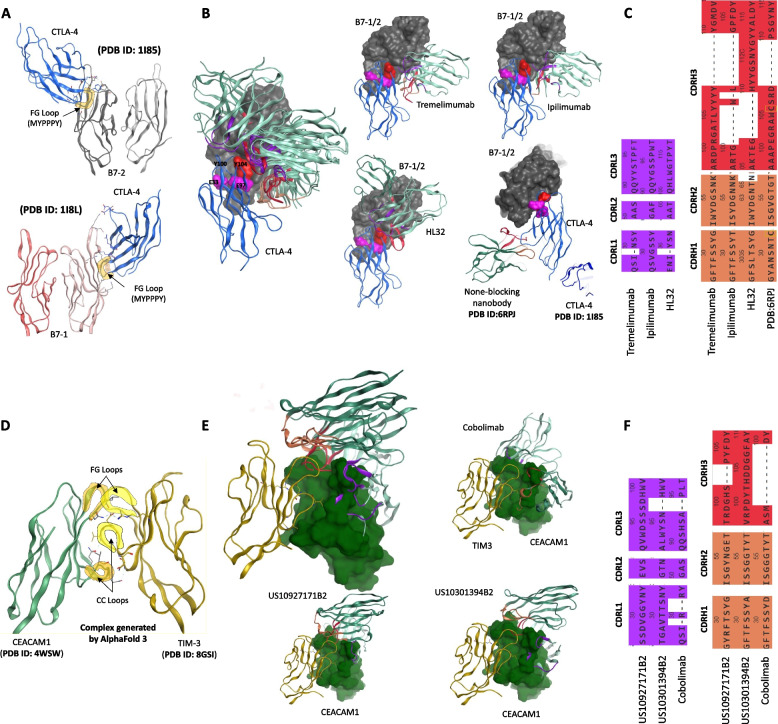
Binding mechanism of CTLA-4 with B7 and TIM-3 with CEACAM1 and immune checkpoint inhibiting monoclonal antibodies (mAbs). **A** Binding interface of CTLA-4 with B7-1 and B7-2 molecules. **B** Epitopes mapping and interface annotation of the CTLA-4 targeting mAbs. **C** Alignment of the complementarity-determining regions (CDRs) from the mAbs presented in panel **B**. **D** Binding mechanism of TIM-3 with CEACAM1. **E** Superimposition of TIM-3 binding mAbs, constructed using AlphaFold 3. **F** Alignment of the CDRs from the mAbs depicted in panel **E**

CTLA-4 binds B7-1 and B7-2, with high affinity, utilizing its FG loop, which contains the highly conserved 99-MYPPPY-104 motif [55]. This motif plays a critical role in mediating interactions between CTLA-4 and B7 molecules by establishing hydrophobic contacts and hydrogen bonds (Table 3). CTLA-4 also forms salt bridges through E33 and E97, interacting with R29 and R94 on B7-1 and K90 and R97 on B7-2, respectively (Fig. 4A). These interactions are essential for the inhibitory function of CTLA-4 and therefore remain the primary epitopes of CTLA-4-blocking antibodies.

**Table 3:** Interface of CTLA-4 with B7 molecules (PDB ID: 1I8L (B7-1); PDB ID: 1I85 (B7-2)). This table corresponds Fig. 4A, showing detailed interface of the CTLA4 with B7-1 and B7-2 complexes

**Bond**	**B7-1**	**CTLA-4**	**Energy**	**Dist**
IH	Arg29	Glu33	− 33.83	2.95
IH	Asp90	Arg35	− 29.19	3.01
IH	Lys89	Asp64	− 20.13	2.88
IH	Lys89	Asp65	− 36.89	2.69
IH	Arg94	Glu97	− 27.04	2.83
H	Tyr31	Met99	− 3.1	2.64
A	Tyr31	Pro102	− 0.9	3.91
H	Gln33	Tyr104	− 6.9	3.05
A	Val83	Tyr104	− 0.6	3.91
H	Lys36	Tyr104	− 0.5	3.41
**Bond**	**B7-2**	**CTLA-4**	**Energy**	**Dist**
IH	Lys90	Glu33	− 36.13	2.68
IH	Arg97	Glu97	− 19.32	2.99
A	Phe31	Pro102	− 0.5	4.18
A	Tyr44	Tyr100	0	3.83
H	Met95	Tyr100	− 1.3	4.37

Unit for energy is kcal/mol

*I* ionic bonds, *H* hydrogen bonds

Two CTLA-4-targeting antibodies, ipilimumab and tremelimumab, have received regulatory approval for the treatment of hepatocellular carcinoma (HCC) in combination regimens [32, 56]. Ipilimumab, combined with nivolumab, has been approved by the FDA for previously treated HCC patients, based on its efficacy in pivotal clinical trials [56]. Similarly, tremelimumab, in combination with durvalumab, demonstrated robust clinical efficacy in patients with unresectable HCC and received FDA approval in 2022 [30] (NCT03298451). This combination therapy leverages the dual mechanisms of immune checkpoint inhibition, where ipilimumab targets and blocks CTLA-4 to boost T-cell activity, while nivolumab inhibits PD-1, together creating a synergistic enhancement of the anti-tumor immune response. This dual inhibition aims to reinvigorate exhausted T-cells, allowing for stronger immune-mediated destruction of tumor cells in HCC. Moreover, tremelimumab and durvalumab, known as the STRIDE (Single Tremelimumab Regular Interval Durvalumab) regimen, have been shown to improve overall survival in clinical trials compared to the standard of care, underscoring the clinical potential of this combination therapy for advanced HCC [57].

Upon superimposing tremelimumab and ipilimumab on the CTLA-4/B7 complex, we observed that both antibodies effectively block the critical 99-MYPPPY-104 motif, along with residues E33 and E97 of CTLA-4, which are essential for B7 binding (Fig. 4B). A third antibody, HL32, which has shown potential in preclinical studies [58, 59], also targets the MYPPPY-containing epitope, though it adopts a slightly different binding orientation compared to tremelimumab and ipilimumab (Fig. 4B, *bottom panel*). A nanobody that does not obstruct the B7 binding interface on CTLA-4 has also been identified (PDB ID: 6RPJ), although its co-structure with CTLA-4 has yet to be resolved. To investigate this further, we used AlphaFold 3 to model the nanobody-CTLA-4-B7 complex, revealing that the nanobody binds to an epitope distinct from the B7 interface, targeting residues outside the regions recognized by tremelimumab and ipilimumab. In addition, this nanobody does not interfere with the CTLA-4 dimerization (Fig. 4B, *bottom panel*) that has been reported earlier [60]. Since the homodimerization of CTLA-4 is essential for its ability to bind B7 molecules with high affinity, disrupting this interaction could prevent CTLA-4-mediated immune suppression without affecting other immune checkpoint functions. Interestingly, while tremelimumab and HL32 feature relatively longer CDRH3 regions, ipilimumab exhibits a shorter CDRH3. Additionally, while the CDRH1 regions of tremelimumab and ipilimumab are nearly identical, the other two CDRs in the VH domain show limited sequence identity (Fig. 4C).

### TIM-3 targeting antibodies in HCC

TIM-3/CEACAM1 complex is crucial for mediating immune regulatory functions, with both proteins playing significant roles in T-cell regulation [61]. According to the previously proposed model, TIM-3/CEACAM1 primarily interacts through their IgV domains, utilizing their FG and C–C loops [62, 63]; however, precise model has not been proposed yet. We modeled the heterodimer of TIM-3/CEACAM1 using AlphaFold 3 and found that FG and C–C loops form the core of the binding interface, engaging in hydrophobic contacts and hydrogen bonding, which are essential for stabilizing the complex (Fig. 4D and Table 4).

**Table 4:** Interface of TIM-3 with CEACAM1 molecule. This table corresponds Fig. 4D, showing detailed interface of the TIM-3 and CEACAM1 complex

Bond	TIM-3	CEACAM1	Energy	Dist
H	Asn65	Val39	− 5.5	2.89
H	Asn65	Arg38	− 4.9	2.77
H	Val60	Gln44	− 4.2	2.79
H	Glu62	Asn97	− 3.8	2.71
H	Asn65	Asp40	− 2.6	2.85
H	Ala57	Asn42	− 2.1	2.95

Unit for Energy is kcal/mol; *H* Hydrogen bonds

Till now, only cobolimab has been evaluated in combination with dostarlimab in clinical trials for advanced HCC [37] (NCT03680508); however, there are multiple other candidates that are investigated preclinically. None of the TIM-3 targeting mAbs or nanobodies has been structurally resolved with or without TIM-3; hence, we modeled cobolimab and two mAbs US10301394B2 [64] and US10927171B2 [65] through AlphaFold 3 together with TIM-3 to investigate their epitope and binding inhibitory mechanisms. Upon superimposing the docked antibodies onto the TIM-3/CEACAM1 structure, we observed that all three antibodies bind to an overlapping epitope that includes both the FG and C–C’ loops of the TIM-3 (Fig. 4E). Notably, these antibodies directly compete with CEACAM1 for binding to TIM-3, thereby potentially disrupting the TIM-3/CEACAM1 interaction. This competitive inhibition by antibodies may underlie their therapeutic efficacy in restoring immune activation by blocking TIM-3’s suppressive effects [37].

## Bispecific antibodies targeting the immune suppressive molecules

Combination therapies with mAbs aimed at distinct receptors or epitopes can boost treatment outcomes and help mitigate resistance to drugs; however, these approaches may lead to increased toxicity [66]. To address this, bispecific antibodies (bsAbs) have been developed to target two distinct antigens or epitopes at once, offering a potential solution to improve both effectiveness and safety. Studies have shown that bsAbs can boost T cell activation and proliferation in the presence of tumor cells, leading to enhanced anti-tumor activity [67–69]. Numerous bsAbs are currently undergoing preclinical and clinical evaluation in cancer treatment [69]. Here, we focus on the structural characteristics of a few bsAbs that simultaneously target PD-1, PD-L1, CTLA-4, and LAG-3 molecules.

**Fig. 5:**
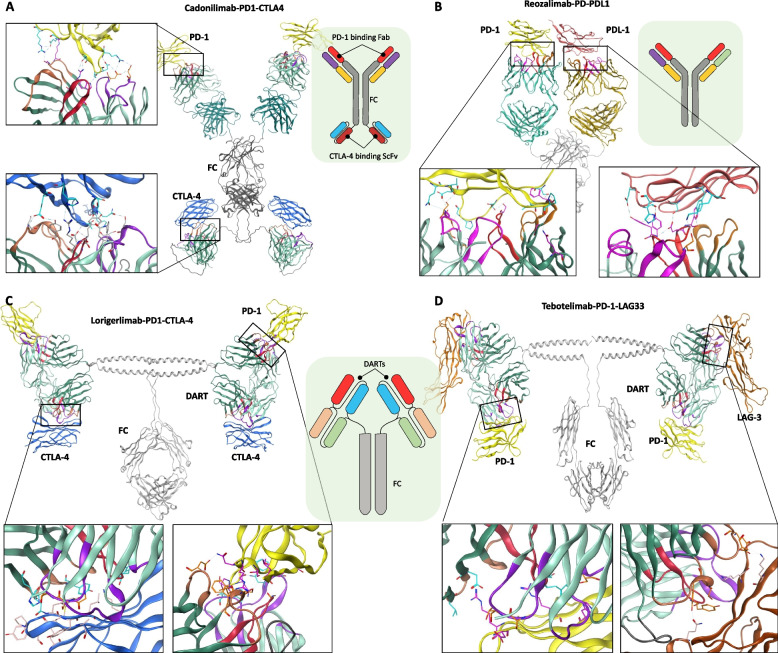
Binding mechanisms and epitope mapping of PD-1, CTLA-4, and LAG-3 inhibiting bispecific antibodies (bsAbs).** A** This panel illustrates the binding mechanism of cadonilimab, a bispecific antibody targeting both CTLA-4 and PD-1.** B** The binding interface of reozalimab, which simultaneously targets PD-L1 and PD-1. **C** This panel details the binding interface of lorigerlimab, which targets both PD-1 and CTLA-4. **D** The binding interface of tebotelimab, a bispecific antibody targeting PD-1 and LAG-3

Cadonilimab (AK104) is a bispecific IgG1 antibody with a tetravalent structure and an Fc-null design, eliminating immune effector functions. Clinical trials showed that it was safe, with low rates of serious irAEs, and effective against tumors. Based on these results, cadonilimab was approved in June 2022 by the China National Medical Products Administration for recurrent or metastatic cervical cancer [70, 71]. In addition, cadonilimab has also been approved in China for HCC [72]. Structurally, cadonilimab employs an Fc-linked scFv domain to target CTLA-4, while its regular IgG Fabs bind PD-1. Computational modeling of cadonilimab-bound PD-1 and CTLA-4 shows engagement of both the light and heavy chain CDRs in target binding (Fig. 5A).

Reozalimab (LY3434172, IBI318) is another bispecific IgG1 antibody with an ablated Fc domain that simultaneously targets PD-1 and PD-L1. Preclinical studies, including in vitro and human xenograft models, have demonstrated better antitumor activity for reozalimab compared to the use of individual mAbs against PD-1 or PD-L1, or their combination [73]. Reozalimab is currently undergoing clinical trials and has demonstrated promising safety and efficacy in patients with advanced-stage cancers (NCT03875157) [74]. Structural analysis using AlphaFold 3 revealed that both Fabs of reozalimab bind specifically to PD-1 and PD-L1, utilizing their VH and VL CDRs without steric hindrance (Fig. 5B).

Lorigerlimab (MGD019) is a bispecific Fc-bearing (IgG4) (dual-affinity re-targeting molecule) DART molecule designed to enhance CTLA-4 blockade on tumor infiltrating lymphocytes, while maintaining maximal PD-1 blockade on PD-1 expressing cells. A DART molecule is an engineered bsAbs designed to simultaneously bind to two different epitopes [75]. DARTs have a distinct structure compared to traditional bsAbs, allowing them to effectively engage two targets and often direct immune system activity, such as cytotoxic T cells, toward a specific target like a tumor cell. Lorigerlimab demonstrates a manageable safety profile with evidence of encouraging and durable antitumor activity in a chemotherapy refractory metastatic castration-resistant prostate cancer (mCRPC) population [76]. Unlike cadonilimab, where the CTLA-4 targeting ScFv molecule is linked with the Fc domain, in lorigerlimab, both PD-1 and CTLA-4 targeting CDRs are located on a single DART molecule, but in opposite orientation (Fig. 5C). Both chains in the Fc domain are provided with extended helical arms to accommodate two DART molecules, providing maximum capacity neutralization. Similar to lorigerlimab, tebotelimab (formerly known as MGD013) is also an IgG4 tetravalent bispecific DART molecule that target both PD-1 and LAG-3 [77] (Fig. 5D). Tebotelimab has also demonstrated satisfactory results in patients with advanced HCC, who had failed prior targeted therapy and/or immunotherapy (NCT04212221) [77].

**Fig. 6:**
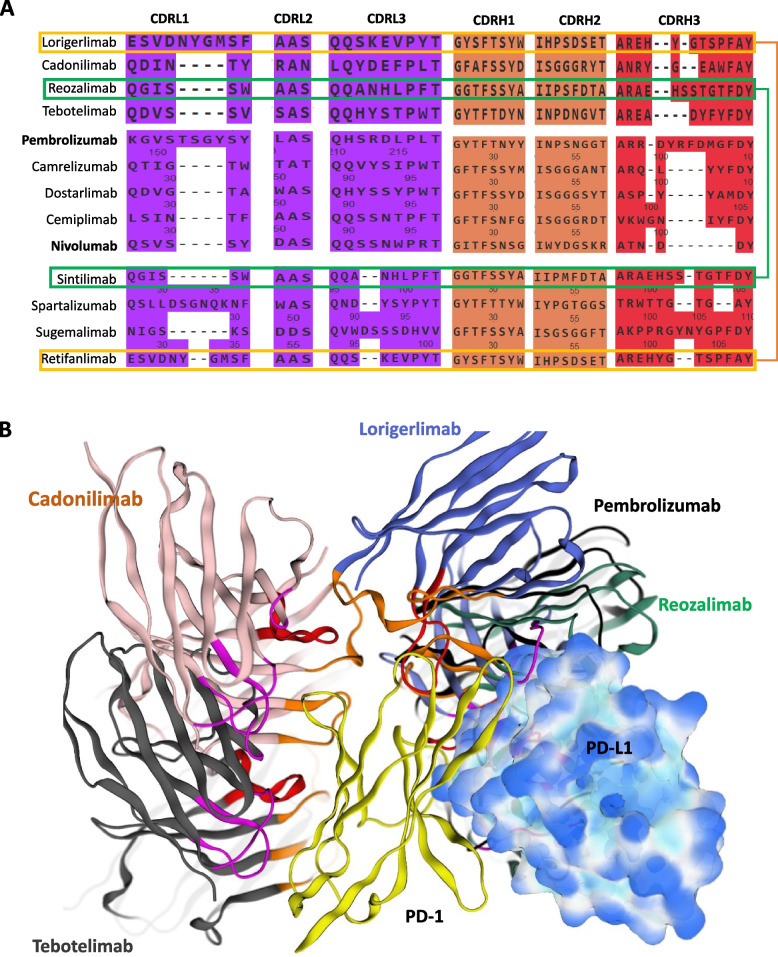
CDR alignment and epitope mapping of PD-1 targeting bispecific antibodies (bsAbs) and monoclonal antibodies (mAbs).** A** Alignment of the heavy and light chain CDRs from PD-1 targeting bsAbs and mAbs, showing two pairs of identical CDRs between the bsAbs and mAbs. **B** The epitopes of lorigerlimab and reozalimab overlap with the PD-L1 interface, as well as with the epitope of pembrolizumab on PD-1

We further examined the CDRs of the four bispecific antibodies (bsAbs) targeting PD-1 and aligned them with those of PD-1-targeting mAbs or nanobodies. Our analysis showed that all six CDRs in lorigerlimab and reozalimab bsAbs were identical to the corresponding CDRs in retifanlimab and sintilimab mAbs, respectively (Fig. 6A). These bsAbs also overlapped with the PD-1 epitope recognized by pembrolizumab, similar to PD-1-targeting mAbs, retifanlimab, and sintilimab shown in Fig. 1. In contrast, the CDRs of cadonilimab and tebotelimab displayed distinct binding patterns to PD-1, targeting epitopes that did not overlap with the binding site of pembrolizumab or the PD-1/PD-L1 interface. These findings suggest that bsAbs with non-overlapping epitopes could be leveraged in combination therapies simultaneously, enhancing therapeutic efficacy while minimizing resistance. This approach has potential for maximizing synergy between different immune checkpoint inhibitors.

## Antibodies targeting the immune suppressive immune cells

As discussed above, the TIME of HCC is characterized by a complex infiltration of immune-suppressing cells such as Tregs, MDSCs, and TAMs (Fig. 1). Tregs play a significant role in maintaining the immunosuppressive environment in HCC by producing cytokines like TGF-β and IL-10, which inhibit effector T-cell responses [78]. Tregs also express high levels of CTLA-4, which further attenuates immune activation [79]. Targeting CTLA-4 with antibodies, such as ipilimumab or tremelimumab, can enhance effector T-cell responses and deplete Tregs in the TME, reducing immunosuppression and enhancing tumor immune surveillance. MDSCs suppress T-cell activity through the production of immunosuppressive cytokines and depletion of key nutrients like arginine [80]. Additionally, they contribute to tumor growth and metastasis by promoting angiogenesis and tissue remodeling. Colony-stimulating factor 1 receptor (CSF-1R) plays a crucial role in the regulation and survival of MDSCs and TAMs in HCC [81]. Antibodies like cabiralizumab [82] and emactuzumab [83], which target CSF-1R, aim to deplete these suppressive cells and reprogram the tumor microenvironment, thereby enhancing anti-tumor immune responses. TAMs are typically polarized toward an M2 phenotype, supporting tumor growth and immunosuppression through the secretion of pro-tumorigenic cytokines such as IL-10, TGF-β, and IL-6 [84, 85]. These cytokines inhibit cytotoxic T cell responses, promote the recruitment of other immunosuppressive cells like Tregs, and foster an environment conducive to tumor progression (Fig. 1). Furthermore, M2-TAMs contribute to angiogenesis by secreting VEGF, which supports the formation of new blood vessels necessary for sustaining tumor growth [86]. Targeting TAM-associated pathways, such as CSF-1R and CCR2, with AMG820 and PF-04136309 antibodies, respectively, can reprogram TAMs toward a pro-inflammatory M1 phenotype, supporting anti-tumor immunity [87]. Such strategies are often combined with other ICIs to enhance therapeutic efficacy by reducing immunosuppression and creating a more favorable immune environment (Table 5).

**Table 5:** Antibodies against immune suppressive immune cells-associated molecules

Antibody	Target	Target cell	Mechanism of action	Clinical status	NCT
Cabiralizumab	CSF-1R	TAMs, MDSCs	Inhibits CSF-1R signaling, reducing the recruitment and suppressive function of TAMs and MDSCs	HCC (trials)	NCT04050462
AMG 820	CSF-1R	TAMs, MDSCs	Reduces recruitment and function of immunosuppressive TAMs and MDSCs	Solid tumors (trials), HCC (none)	NCT02713529
Bevacizumab	VEGF	TAMs, MDSCs	Inhibits VEGF to reduce angiogenesis and TAM-mediated immunosuppression	HCC (approved in combo with atezolizumab), Colorectal (approved)	NCT03434379 (HCC)
Monalizumab	NKG2A	NK, T cells	Blocks NKG2A to restore NK and T cell activity against tumors	SCC (trials), HCC (none)	NCT02643550
Relatlimab	LAG-3	Tregs, exh-T cells	Blocks LAG-3 to enhance T cell activation and reduce Treg-mediated suppression	Melanoma (approved), HCC (trials)	NCT03470922 NCT05337137 (HCC)
Etigilimab (MPH313)	TIGIT	Tregs, exh-T cells	Blocks TIGIT to restore T cell activity and reduce Treg-mediated suppression	Solid tumors (trials), HCC (none)	NCT04761198
PF-04136309	CCR2	TAMs, MDSCs	Inhibits CCR2 signaling, reducing recruitment and function of TAMs and MDSCs	PDAC (trials), HCC (none)	NCT02732938
Fresolimumab	TGF-β	CAFs	Neutralizes TGF-β, reducing its immunosuppressive effects	NSCLC (trials), HCC (none)	NCT02581787
NIS793	TGF-β	CAFs	Neutralizes TGF-β, reducing its immunosuppressive effects	Advanced tumors (trials)	NCT02947165
Pamrevlumab	CTGF	CAFs	Inhibits CTGF, a key regulator of fibrosis, reducing tumor stiffness and enhancing immune infiltration	Pancreatic cancer (trials), HCC (none)	NCT02210559
RO6874281 Sibrotuzumab	FAP	CAFs	Targets FAP-expressing CAFs, which are involved in ECM remodeling and immunosuppression	PDAC (trials), HCC (none)	NCT03193190

CAFs play a pivotal role in establishing an immunosuppressive TME by secreting various cytokines and growth factors, including TGF-β, IL-6, and VEGF [88]. TGF-β is particularly critical, as it suppresses the activity of cytotoxic T-cells, promotes the differentiation of Tregs, and inhibits the differentiation and function of Th1 cells and NK cells [89]. Antibodies like fresolimumab and NIS793, which target TGF-β, are being investigated in several clinical trials (Table 5). CAFs also recruit additional immunosuppressive cells, such as MDSCs, TAMs, and Tregs, by secreting chemokines such as CCL2, CCL5, and CXCL12 [90]. Therapies targeting CAF-associated pathways are being explored to counteract their effects. For instance, pamrevlumab, targeting connective tissue growth factor (CTGF) expressed by CAFs, aims to reduce tumor fibrosis and stiffness, thereby enhancing immune cell infiltration and promoting anti-tumor activity [91, 92]. Similarly, antibodies, sibrotuzumab [93] and [94], targeting fibroblast activation protein (FAP), modulate CAF activity, inhibit ECM remodeling, and diminish immunosuppression within the TME [95]. These therapeutic strategies hold promise for dismantling the CAF-induced immunosuppressive barrier, making tumors more susceptible to immune-mediated destruction.

The strategic targeting of immunosuppressive elements within the TIME is essential for enhancing the efficacy of current immunotherapies in HCC. By combining immune ICIs with agents that deplete Tregs, reprogram TAMs, or block MDSCs and CAFs, we can disrupt the intricate networks of immunosuppression that protect tumors from immune destruction. Such a multifaceted approach aims to reinvigorate the immune system’s ability to recognize and eliminate tumor cells more effectively.

## Conclusions

Advancements in antibody-based immunotherapies have significantly reshaped the treatment landscape for HCC, yet several challenges still limit the full potential of these therapies. One of the primary obstacles stems from the highly immunosuppressive TME. Additionally, the risk of hepatic toxicity, especially when using combination therapies, highlights the need for balancing treatment efficacy with minimizing liver damage. Cancer therapeutics are moving toward multi-modal strategies designed to overcome these barriers. A promising direction involves combining ICIs with TKIs, anti-VEGF agents, and other targeted therapies to address various aspects of tumor biology. Early results from ongoing clinical trials suggest that these combinations are improving tumor control and potentially increasing survival outcomes for HCC patients. Beyond these combinations, the development of conjugated antibodies and bi-specific antibodies offers exciting potential. Antibody–drug conjugates combine the targeting specificity of antibodies with the potent cytotoxicity of linked drugs, delivering therapeutic agents directly to tumor cells.

In conclusion, while resistance and toxicity remain challenges, the future of HCC treatment lies in the continued development of combination therapies, biomarker-driven personalization, and next-generation immunotherapies like conjugated and bi-specific antibodies. Ongoing research and clinical trials are expected to refine these approaches, offering more effective, durable treatment options, ultimately improving survival rates and quality of life for HCC patients.

## Computational tools and servers utilized

In this study, the RCSB PDB database was primarily used to retrieve the available protein structures. In cases where the protein structures were unavailable, we utilized AlphaFold software, its server, and database to generate 3D models. For predicting antibody-antigen (Ab-Ag) complexes, we employed AlphaFold 3 server, AlphaFold multimers module, ZDOCK, and ClusPro. However, we predominantly relied on the models suggested by AlphaFold. For protein structure visualization and interface analysis, the MOE suite was used.

## Data Availability

No datasets were generated or analysed during the current study.
